# Case report of retroperitoneal ectopic pancreas with adrenal adenoma

**DOI:** 10.3389/fsurg.2022.935211

**Published:** 2022-09-07

**Authors:** Zhaochen Sun, Tao Chen, Xuefeng Zhu, Jie Geng, Chaofan Sui, Nan Zhang, Lingfei Guo

**Affiliations:** ^1^ Graduate School, Shandong First Medical University and Shandong Academy of Medical Sciences, Jinan, China; ^2^ Clinical Laboratory, Jinan Maternal and Child Care Hospital, Jinan, China; ^3^ Department of Radiology, Zouping People's Hospital of Shandong Province, Zouping, China; ^4^ Department of Director's Office, Jinan Municipal Health Commission, Jinan, China; ^5^ Department of Radiology, Shandong Provincial Hospital Affiliated to Shandong First Medical University, Jinan, China

**Keywords:** ectopic pancreas, retroperitoneum, adrenal adenoma, accessory spleen, magnetic resonance imaging, imaging features

## Abstract

**Background:**

Ectopic pancreas is a congenital anomaly in which pancreatic tissue is anatomically separated from the main gland and without vascular or ductal continuity. A case of retroperitoneal ectopic pancreas with adrenal adenoma has never yet been reported.

**Case Presentation:**

A 54-year-old man presented three masses in the left retroperitoneum, and two of them were resected. The pathologic findings were a retroperitoneal ectopic pancreas with adrenal adenoma.

**Conclusion:**

We report an extremely rare case of a retroperitoneal ectopic pancreas and its characterization with dynamic gadolinium-enhanced magnetic resonance imaging (MRI).

## Background

An ectopic pancreas is a congenital anomaly in which pancreatic tissue is anatomically separated from the main gland and without vascular or ductal continuity (1). Autopsy results indicate a prevalence of 0.55%–13.7%, and the condition is estimated to be incidentally encountered during 0.2% of upper abdominal surgeries and 0.9% of gastrectomies (2, 3). Ectopic pancreas occurs in the stomach (25%–52%), duodenum (27%–36%), and jejunum (15%–17%). Less common sites of an ectopic pancreas include the ileum, esophagus, and Meckel's diverticulum, but it can occasionally occur in the mesentery, hepatobiliary system, spleen, mediastinum, lung, and umbilical foramina (1, 4). We report a case of a retroperitoneal ectopic pancreas with ipsilateral adrenal adenoma and accessory spleen.

## Case presentation

A 54-year-old man presented with “left adrenal area occupation” on medical examination. Laboratory tests showed neuron-specific enolase elevation (NSE) of 20.00. Renin, aldosterone (ALD), cortisol (COR), aldosterone/renin concentration ratio (ARR), angiotensin II, potassium, sodium, and chlorine were within the normal range, and blood pressure was 150/103 mmHg. No positive signs were found in the abdomen during physical examination.

### Imaging examinations

**Figure 1:**
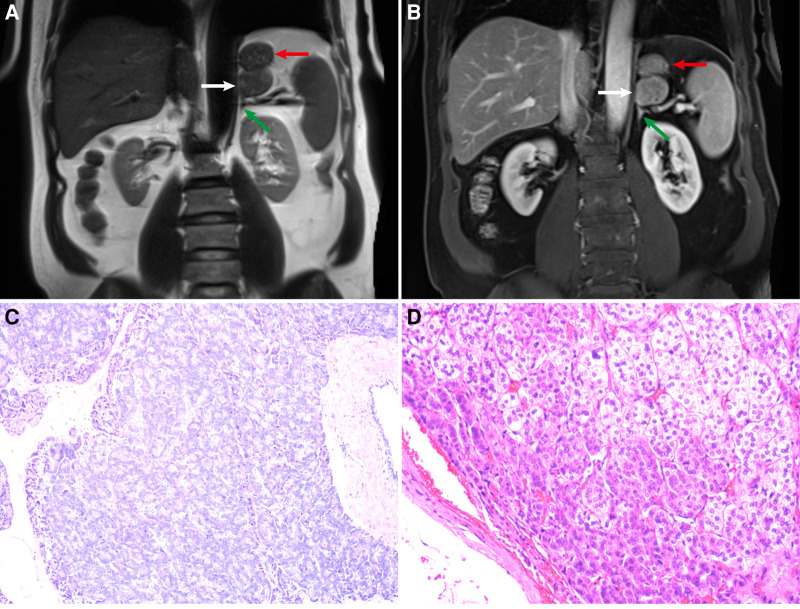
(**A**) Coronal T2-weighted image (T2WI). (**B**) Coronal contrast-enhanced fat-suppressed T1-weighted MR image (FS-T1WI). The white, red, and green arrows refer to the ectopic pancreas, stomach, adrenal gland, respectively. (**C**) Ectopic pancreas (hematoxylin–eosin stain; original magnification ×100). (**D**) Adrenal cortical adenoma (hematoxylin–eosin stain; original magnification ×100).

**Figure 2:**
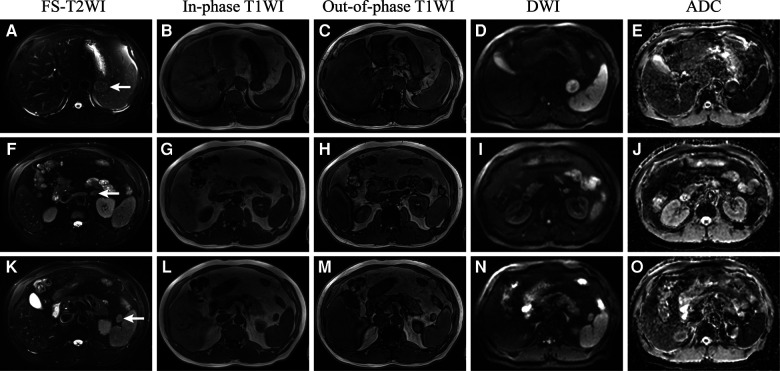
Retroperitoneal ectopic pancreas (**A**–**E**), adrenal adenoma (**F**–**J**), and accessory spleen (**K**–**O**) in FS-T2WI (fat suppressed T2WI), in-phase T1WI, out-of-phase T1WI, diffusion-weighted imaging (DWI), and apparent diffusion coefficient (ADC).

Enhanced MRI (Figure 1B) of the upper abdomen revealed a mass (3.5 × 3.0 cm) between the stomach and the left adrenal gland. The mass was isointense in the T1-weighted image (T1WI) (Figure 2B) and isointense in the T2-weighted image (T2WI) (Figure 1A). In the out-of-phase T1WI (Figure 2C), small patches of decreased signals were observed. DWI and ADC (Figures 2D, E) showed limited diffusion, the ADC value was 1001.4, and enhanced scanning showed uneven delayed enhancement. A round mass (1.6 × 1.2 cm) with slight hypointensity on T1WI and isointensity on fat-suppressed T2WI (FS-T2WI) was found in the inferior part of the left adrenal gland (Figures 2F–J). The signal in the out-of-phase T1WI was obviously lower than that in the in-phase T1WI. Contrast-enhanced MRI of the mass showed delayed enhancement. A signal of 1.7 × 1.5 cm in cross-section was observed in the splenic hilum, which was consistent with that of the spleen (Figures 2K–O). The characteristics of the ectopic pancreas, pancreas, adrenal adenoma, and accessory spleen are presented in Table 1. The descriptions of these masses in fat-suppressed T2-weighted imaging (FS-T2WI), in-phase T1WI, out-of-phase T1WI, and diffusion-weighted imaging (DWI) were compared with those of the liver. The values of the ADC and the dynamic enhanced fat-suppressed T1-weighted MR image (FS-T1WI) were recorded by ITK-SNAP (www.itksnap.org).

**Table 1:** MRI characteristics of the ectopic pancreas, pancreas, adrenal adenoma, and accessory spleen.

	FS-T2WI	In-phase T1WI	Out-of-phase T1WI	DWI	ADC (10^−6 ^mm^2^/s)	FS-T1WI^a^	Arterial phase^a^	Venous phase^a^	Delay phase^a^
Ectopic pancreas	Slightly hyperintensity	Slightly hypointensity	Isointensity	Hyperintensity	1001.4	76.6	265.4	526.8	519.3
Pancreas	Isointensity	Isointensity	Isointensity	Slightly hyperintensity	1105.3	254.2	596.3	474.0	392.5
Adrenal adenoma	Isointensity	Slightly hypointensity	Hypointensity	Hyperintensity	540.3	227.2	397.0	343.0	265.1
Accessory spleen	Slightly hyperintensity	Slightly hypointensity	Isointensity	Slightly hyperintensity	1483.3	196.4	687.6	607.3	474.0

^a^ The values measured by ITK-SNAP express the intensity of the signal.

### Surgical findings and pathological examination results

Based on these findings, the resection of retroperitoneal masses was performed. Under general anesthesia, the perirenal fat was separated, and the adrenal mass, approximately 2 cm in diameter, was bluntly excised. The retroperitoneal mass was observed along the splenic pedicle vessels, with a diameter of approximately 3 cm. The mass adhered to the splenic pedicle vessels, pancreas, and other surrounding tissues and was removed by an ultrasonic knife after blunt separation. The postoperative course was uneventful, and the patient was discharged 7 days after surgery.

The pathology of the retroperitoneal mass was pancreatic tissue (Figure 1C). By light microscopy examination of HE-stained tissue sections, it was observed that pancreatic acini were composed of tubular glands. The nuclei of acinar cells were round and located at the base of the cells. The cytoplasm contained rich granules, and small ducts were seen between the acini. The pathology of this mass in the inferior part of the left adrenal gland was an adrenal cortical adenoma (Figure 1D). Microscopic examination of the lesion showed that the tumor cells were arranged in funicular and nests. Two types of tumor cells can be seen, one close to the capsule with acidophilic cytoplasm and the other far from the capsule with clear, lipid-rich cytoplasm. Two types of tumor cells had mild atypia and no pathological mitotic figure.

## Discussion

Ectopic pancreas is more common in elderly individuals, with the peak of morbidity between 40 and 60 years, and is more common among males (3). It is rare to find an ectopic pancreas in the retroperitoneum. We searched the keywords retroperitoneal ectopic pancreas in PubMed and found two reports. The masses of the retroperitoneal ectopic pancreas ;ported by Lin et al. were similar to bilateral adrenal tumors (5). The ectopic pancreas reported by Moriki et al. was a lipomatous mass with a fatty component predominating on imaging (6).

The precise pathogenic mechanism of ectopic pancreas in humans is poorly understood (7). Migration theory holds that during foregut rotation, some pancreatic tissue fragments are separated from the main body and deposited in abnormal locations (1, 7). During embryonic development, part of the pancreatic tissue falls into the retroperitoneum and established an independent blood supply, which may explain the occurrence of retroperitoneal ectopic pancreas.

Preoperative diagnosis of the ectopic pancreas is difficult. It is usually asymptomatic, but depending on size, location, and pathological changes, it may become clinically evident (2, 4, 8). The most common clinical symptom of the ectopic pancreas is epigastric pain (3). In this case, there were no obvious clinical signs, only elevated blood pressure. We initially considered that hypertension might be related to the excessive secretion of aldosterone or cortisol caused by an adrenal adenoma (9), but laboratory results showed that both of these indicators were normal; therefore, we assumed primary hypertension to be the cause.

For ectopic pancreas, MRI has high soft tissue resolution (10). Dynamic enhanced MRI can better display the blood supply characteristics of the masses. By analyzing specific sequence signals, the components of the masses can be analyzed, such as lipids, fats, and water. We can see a circular mass indistinguishable from the stomach and the left adrenal gland in the coronal T2-weighted image (Figure 1A). Small gaps seemed to be found between the mass and the stomach or adrenal glands on coronal contrast-enhanced FS-T1WI (Figure 1B). Therefore, the mass was considered to have originated in the retroperitoneum. In the inferior part of the left adrenal gland, the low-intensity mass in out-of-phase T1WI was considered an adrenal adenoma (9, 11). In the splenic hilar region, the mass with the same signal as the spleen was considered an accessory spleen.

**Figure 3:**
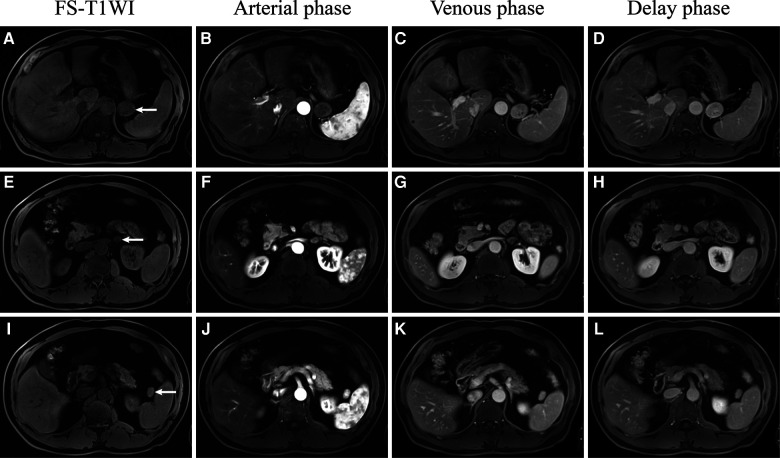
Dynamic gadolinium-enhanced MRI of the retroperitoneal ectopic pancreas (**A**–**D**), adrenal adenoma (**E**–**H**), and accessory spleen (**I**–**L**).

MRI signals of the ectopic pancreas should be similar to those of the normal pancreas (1,1,22). However, in this case (Figure 3; Table 1), the intensity of the ectopic pancreas was lower than that of the normal pancreas in the T1WI. In addition, Okuhata et al. believed that dynamic imaging clearly showed the synchronicity of the imaging results of an ectopic pancreas and normal pancreas (12). The ectopic pancreas, in this case, showed delayed enhancement (Figures 3A–D; Table 1), while the normal pancreas showed high intensity in the delayed arterial phase. The reason may be related to different blood supplies. Moreover, the ectopic pancreas showed stronger enhancement in the venous and delayed phases, which was associated with the presence of a large number of acini. The predominantly acini-dominant type showed a stronger enhancement than the other types (13). Pancreatic ectopic tissue composed of acinar cells, islet cells, conduit cells, etc., causes inhomogeneity in the image. Sometimes ductal structures can be seen in the upper gastrointestinal ectopic pancreas (1, 4), but we did not find them in this retroperitoneal ectopic pancreas.

The malignancy rate of retroperitoneal masses is not low. For this case of retroperitoneal mass, we decided to remove the lesion; although the imaging manifestations seem to be benign, the volume is relatively large. We were confident in the diagnosis of adrenal adenoma and that it would be convenient to remove the adrenal adenoma at the same time as surgery, and we decided to perform an excision of the two retroperitoneal masses. The pathological results were “retroperitoneal ectopic pancreatic” and “adrenal adenoma.” Ectopic pancreatic tissue is susceptible to the same pathological conditions that affect the *in situ* pancreas, including pancreatitis, pseudocyst formation, and benign and malignant tumors (2, 4). Malignant transformation of ectopic pancreatic tissue is a rare event and a diagnostic challenge, as the clinical symptoms and radiographic features of these tumors are nonspecific (14). It would be prudent to resect ectopic pancreases surgically upon discovery (15). After 18 months of follow-up, our patient had no signs of recurrence or metastasis, and imaging follow-up is still needed.

## Conclusion

In summary, we report a rare case of a retroperitoneal ectopic pancreas with adrenal adenoma and accessory spleen. A review of the literature did not find any correlation between the pathogenesis of the three diseases. MRI findings of the retroperitoneal ectopic pancreas were different from those of the previously found ectopic pancreas.

## Data Availability

The raw data supporting the conclusions of this article will be made available by the authors, without undue reservation.

## References

[1] Rezvani M Menias C Sandrasegaran K Olpin JD Elsayes KM Shaaban AM. Heterotopic pancreas: histopathologic features, imaging findings, and complications. Radiographics. (2017) 37:484–99. 10.1148/rg.2017160091 28287935

[2] Kung JW Brown A Kruskal JB Goldsmith JD Pedrosa I. Heterotopic pancreas: typical and atypical imaging findings. Clin Radiol. (2010) 65:403–7. 10.1016/j.crad.2010.01.005 20380941

[3] Wei R Wang Q Chen Q Liu J Zhang B. Upper gastrointestinal tract heterotopic pancreas: findings from CT and endoscopic imaging with histopathologic correlation. Clin Imag. (2011) 35:353–9. 10.1016/j.clinimag.2010.10.001 21872124

[4] Yang C Che F Liu X Yin Y Zhang B Song B. Insight into gastrointestinal heterotopic pancreas: imaging evaluation and differential diagnosis. Insights Imaging. (2021) 12:144–13. 10.1186/s13244-021-01089-0 34674040 PMC8531187

[5] Lin L Ko S Huang C Ng S Lin J Sheen-Chen S. Retroperitoneal ectopic pancreas: imaging findings. Br J Radiol. (2009) 82:e253–5. 10.1259/bjr/27696141 19934067 PMC3473376

[6] Moriki T Ohtsuki Y Takahashi T Ueta S Mitani M Ichien M Lipoma-like tumor mass probably arising in the retroperitoneal heterotopic pancreas: a previously undescribed lesion. Pathol Int. (2004) 54:527–31. 10.1111/j.1440-1827.2004.01661.x 15189508

[7] Rodríguez Seguel E Villamayor L Arroyo N De Andrés MP Real FX Martín F Loss of GATA4 causes ectopic pancreas in the stomach. J Pathol. (2020) 250:362–73. 10.1002/path.5378 31875961

[8] Trifan A Tarcoveanu E Danciu M Hutanasu C Cojocariu C Stanciu C. Gastric heterotopic pancreas: an unusual case and review of the literature. J Gastrointestin Liver Dis. (2012) 21:209–12. PMID: 22720312

[9] Low G Sahi K. Clinical and imaging overview of functional adrenal neoplasms. Int J Urol. (2012) 19:697–708. 10.1111/j.1442-2042.2012.03014.x 22462796

[10] Kotlyarov PM. Magnetic resonance imaging in recognition of ectopic pancreatic tissue (clinical observation). Terapevt Arkh. (2018) 90:94–7. 10.26442/terarkh201890294-97 30701782

[11] Elbanan MG Javadi S Ganeshan D Habra MA Rao Korivi B Faria SC Adrenal cortical adenoma: current update, imaging features, atypical findings, and mimics. Abdom Radiol. (2020) 45:905–16. 10.1007/s00261-019-02215-9 31529204

[12] Okuhata Y Maebayashi T Furuhashi S Abe K Takahashi M Kanamori N Characteristics of ectopic pancreas in dynamic gadolinium-enhanced MRI. Abdom Imaging. (2010) 35:85–7. 10.1007/s00261-008-9491-6 19048331

[13] Kim JY Lee JM Kim KW Park HS Choi JY Kim SH Ectopic pancreas: CT findings with emphasis on differentiation from small gastrointestinal stromal tumor and leiomyoma1. Radiology. (2009) 252:92–100. 10.1148/radiol.2521081441 19561251

[14] Cazacu IM Luzuriaga Chavez AA Nogueras Gonzalez GM Saftoiu A Bhutani MS. Malignant transformation of ectopic pancreas. Digest Dis Sci. (2019) 64:655–68. 10.1007/s10620-018-5366-z 30415408

[15] Mundackal N Arslan ME Decker C Lee H Nigam A. The removal of ectopic pancreas to prevent carcinoma development. Am J Surg. (2021) 222:1196–7. 10.1016/j.amjsurg.2021.07.002 34256929

